# Colorectal Cancer Surgery: Laparoscopic vs. Robotic Approaches—A Review of the Literature

**DOI:** 10.3390/jcm15135164

**Published:** 2026-07-02

**Authors:** Raul Mihailov, George Țocu, Gabriel Valeriu Popa, Oana Mariana Mihailov, Adrian Beznea, Bogdan Mihnea Ciuntu, Valerii Luțenco

**Affiliations:** 1 Faculty of Medicine and Pharmacy, Research Centre in the Medical-Pharmaceutical Field, “Dunarea de Jos” University of Galati, 800008 Galati, Romania; raul.mihailov@ugal.ro (R.M.); gabriel.popa@ugal.ro (G.V.P.); oanamihailov@yahoo.com (O.M.M.); adrianbeznea@hotmail.com (A.B.); valerii.lutenco@ugal.ro (V.L.); 2 “Sf. Apostol Andrei” County Emergency Clinical Hospital, 800578 Galati, Romania; 3 Department of General Surgery, Faculty of Medicine, Grigore T. Popa University of Medicine and Pharmacy, 700115 Iasi, Romania; bogdanmciuntu@yahoo.com; 4 General Surgery Clinic, “St. Spiridon” County Emergency Clinical Hospital, 700111 Iasi, Romania

**Keywords:** colorectal cancer, robotic surgery, laparoscopic surgery, robotic colectomy, minimally invasive surgery, intracorporeal anastomosis, oncological outcomes

## Abstract

**Background**: Minimally invasive surgery has become the standard of care in colorectal cancer management, with laparoscopic techniques widely adopted due to their established short-term benefits and comparable oncological outcomes to open surgery. More recently, robotic-assisted surgery has emerged as an advanced minimally invasive alternative, offering enhanced visualization, improved instrument dexterity, and superior ergonomics. However, the extent to which these technical advantages translate into clinically meaningful improvements remains a subject of ongoing debate. **Methods**: A systematic review of the literature was conducted using PubMed, Scopus, and Web of Science databases, including studies published between 2005 and 2025. Eligible studies comprised randomized controlled trials, observational studies, cohort studies, and meta-analyses comparing laparoscopic and robotic colectomy for colon cancer. Outcomes of interest included intraoperative parameters (operative time, blood loss, conversion rate), postoperative outcomes (length of hospital stay, complications, mortality), and oncological endpoints (lymph node yield, resection margins, survival). The review was performed in accordance with PRISMA 2020 guidelines. **Results**: A total of 150 studies met the inclusion criteria. Robotic colectomy was consistently associated with reduced intraoperative blood loss, lower conversion rates to open surgery, and shorter length of hospital stay, albeit at the expense of longer operative times, particularly during the learning phase. Oncological outcomes, including lymph node harvest and margin status, were comparable between approaches, with some studies reporting a modest increase in lymph node yield in robotic procedures. The adoption of intracorporeal anastomosis was significantly higher in robotic surgery, contributing to improved postoperative recovery and reduced wound-related complications. Composite quality metrics, such as textbook outcome (TO), were more frequently achieved in robotic cohorts, largely driven by shorter hospitalization and lower complication rates. However, evidence from randomized controlled trials remains limited, and heterogeneity among studies persists. **Conclusions**: Robotic colectomy and rectal resection represent a safe and effective alternative to laparoscopic surgery in the treatment of colon cancer, offering potential advantages in perioperative outcomes and surgical precision. Its benefits appear particularly relevant in technically demanding cases, such as deep pelvic dissection and obese patients. Nevertheless, higher costs, longer operative times during the initial learning curve, and limited high-quality randomized evidence warrant cautious adoption. Future large-scale randomized studies are needed to clarify long-term oncological outcomes, cost-effectiveness, and the optimal integration of robotic platforms into standard colorectal surgical practice.

## 1. Introduction

Colon cancer remains a leading cause of cancer-related mortality worldwide [1,2]. Advances in surgical techniques have enabled a paradigm shift from open procedures to minimally invasive approaches, including laparoscopic surgery and, more recently, robotic-assisted surgery. The primary objective of surgical management in colon cancer is complete oncologic resection with negative margins, accompanied by adequate lymphadenectomy, preservation of gastrointestinal function, and minimization of perioperative morbidity [3,4].

Despite a decline in incidence attributable to national screening programs, colorectal cancer continues to represent a major global health burden. It is currently the third-most commonly diagnosed malignancy in both sexes, accounting for approximately 10% of all cancers, and ranks second in cancer-related mortality, contributing to 9.4% of deaths worldwide [5].

In the United States, colorectal cancer (CRC) is the second-most commonly diagnosed malignancy and the third leading cause of cancer-related mortality [1,6]. Over the past two decades, minimally invasive surgery—encompassing both laparoscopic and robotic techniques—has become the standard of care in CRC management, largely due to well-documented benefits such as reduced postoperative pain, decreased intraoperative blood loss, and accelerated postoperative recovery [7,8,9].

Data from the ACGME (Accreditation Council for Graduate Medical Education) national operative case log for general surgery residents demonstrated a substantial increase in the adoption of minimally invasive techniques in colorectal surgery, rising from 8% in 2003 to 43% in 2018 [10]. This trend has been supported by robust evidence demonstrating the superiority of laparoscopic over open surgery, with advantages including faster recovery, reduced postoperative pain, shorter hospital stay, and equivalent oncological outcomes [11,12,13].

General surgery has emerged as the principal domain for the application of robotic technologies, with a reported 24-fold increase in utilization since 2010 [14]. Nevertheless, robotic surgery remains less widely adopted than laparoscopy, primarily due to higher associated costs, prolonged operative times, and the requirement for dedicated training. Current evidence comparing perioperative outcomes between robotic and laparoscopic approaches remains limited and, in many instances, conflicting—particularly in the context of low colorectal resections [15,16,17,18].

A significant limitation of the existing literature is the aggregation of heterogeneous colorectal procedures—such as right hemicolectomy, left-sided resections, and low anterior resections—within the same analytical frameworks, potentially obscuring procedure-specific outcomes [19,20]. Moreover, there is a paucity of data evaluating the impact of robotic surgery on composite endpoints such as textbook outcome (TO), which integrate postoperative complications, length of hospital stay, and mortality into a single clinically relevant metric [21,22,23].

Robotic surgical systems have been introduced to address several inherent limitations of laparoscopy, providing enhanced three-dimensional visualization, improved camera stability, superior instrument articulation, tremor filtration, and optimized surgeon ergonomics [24]. Consequently, robot-assisted colorectal surgery has gained increasing acceptance, despite the absence of unequivocal evidence demonstrating clear superiority over conventional laparoscopy [9,25,26,27,28,29,30].

Most comparative studies to date are limited by single-center designs, relatively small sample sizes, and heterogeneous patient populations lacking appropriately matched control groups [31,32].

Although neoadjuvant chemoradiotherapy has enabled 20–35% of patients with rectal cancer to achieve a complete pathological response [33], often followed by carefully selected “watch and wait” or “active surveillance” strategies [34,35], surgical resection remains the cornerstone of curative treatment for both colon and rectal cancer. Over recent decades, surgical practice has progressively shifted from conventional open approaches to advanced laparoscopic techniques, driven by well-established benefits including reduced tissue trauma, attenuated physiological stress response, decreased postoperative pain, earlier mobilization, shorter hospitalization, and improved cosmetic outcomes without compromising long-term oncological efficacy. Accordingly, minimally invasive surgery is currently regarded as the gold standard. However, laparoscopic techniques remain highly operator-dependent, particularly with respect to the experience of the surgical team and camera operator, and are frequently limited by unstable visualization and challenges in maintaining optimal exposure.

The initially high costs of robotic surgery, coupled with the lack of clearly demonstrated advantages in early studies, have limited its widespread adoption. However, a recent resurgence in robotic surgery, driven by rapid technological advancements, has redefined it as an increasingly sophisticated minimally invasive modality. This evolution is reflected in the exponential growth of scientific publications addressing robotic applications in colorectal surgery and other anatomically challenging fields. Despite the absence of haptic feedback, robotic approaches have consistently been shown to be safe and non-inferior to laparoscopic techniques [36].

Importantly, robotic platforms offer several distinct technical advantages: tremor elimination, surgeon-controlled high-definition three-dimensional visualization, and wristed instruments that enhance dexterity and ergonomics. These features facilitate precise dissection in anatomically restricted operative fields while reducing surgeon fatigue and the risk of work-related musculoskeletal disorders [37,38,39]. As a result, robotic systems are more frequently employed in rectal surgery than in colonic procedures. With increasing cumulative experience and the introduction of advanced platforms such as the Da Vinci Xi system, surgeons have expanded indications to include right-sided resections [26,40] and complex multi-quadrant procedures for both benign and malignant conditions.

In such contexts, improved short-term outcomes have been reported, particularly among patients with elevated body mass index (BMI), high operative risk, and advanced disease, including lower conversion rates and improved circumferential resection margins [41,42]. However, these findings predominantly originate from high-volume tertiary centers with substantial expertise, limiting their generalizability. Evidence supporting the implementation of robotic colorectal surgery in lower-volume settings, particularly district general hospitals, remains scarce.

We agree with studies that suggest that robotic platforms may facilitate wider adoption of a completely minimally invasive approach to right hemicolectomy, particularly through the routine implementation of intracorporeal anastomosis. This may translate into improved postoperative recovery compared with the current standard of laparoscopic right hemicolectomy with extracorporeal anastomosis.

Furthermore, robotic right hemicolectomy may accelerate the return of bowel function and consequently reduce hospital length of stay. It may also decrease the incidence of wound-related complications, including incisional hernia, by enabling specimen extraction through non-midline incisions.

## 2. Materials and Methods

For the purpose of this review, a systematic search was conducted across the PubMed, Scopus, and Web of Science databases using the following keywords: colon cancer, robotic colectomy, laparoscopic colectomy, colorectal surgery, minimally invasive surgery. Articles published between 2005 and 2025, in English, reporting clinical and oncological outcomes comparing laparoscopic and robotic colectomy were considered eligible. Randomized controlled trials (RCTs), observational studies, cohort studies, and meta-analyses were included. Following screening for relevance, methodological quality, and sample size, a total of 150 studies were ultimately selected, providing data on complication rates, lymph node yield, transfusion requirements, postoperative survival, and time to initiation of adjuvant chemotherapy.

The systematic review was conducted and reported in accordance with the PRISMA 2020 (Preferred Reporting Items for Systematic Reviews and Meta-Analyses) guidelines [43]. The MEDLINE database was searched without time restrictions for observational studies and randomized clinical trials published in English comparing laparoscopic and robotic colectomy in terms of intraoperative outcomes (such as conversion rate) and/or postoperative outcomes (including length of hospital stay, time to first bowel movement, morbidity, and 30-day mortality). Additional records were identified through manual screening of reference lists from relevant secondary analyses.

The ACS-NSQIP program represents a validated, risk-adjusted, outcomes-based national registry designed to monitor and improve the quality of surgical care by capturing postoperative complications within the first 30 days following surgery. The database includes over 250 variables, encompassing demographic characteristics, preoperative risk factors, intraoperative parameters, and 30-day postoperative morbidity and mortality. Standardized definitions, regular audits, periodic reviews, and annual certification of data abstractors ensure data accuracy and reliability [44].

## 3. Results

A total of 250 articles were initially identified. Following abstract screening, 200 articles were retained, and after full-text evaluation, 170 studies met the inclusion criteria. Ultimately, 150 articles addressing the topic of interest were included in the present analysis. The literature search and study selection process according to PRISMA 2020 is summarized in Table 1.

Reasons for full-text exclusion included lack of direct comparison between robotic and laparoscopic colectomy, insufficient reporting of perioperative or oncological outcomes, duplicate patient populations, and studies with inadequate methodological quality.

### 3.1. Laparoscopic Approach

Laparoscopic colectomy, introduced in the late 1990s, has demonstrated multiple advantages over open surgery, including reduced postoperative pain, faster recovery, and shorter hospital stay [45,46]. Several multicenter studies and randomized controlled trials have shown that laparoscopy provides oncological outcomes comparable to open surgery, with similar rates of circumferential margin positivity and lymph node harvest [47,48].

However, laparoscopy presents limitations in cases involving tumors located deep within the pelvis or in obese patients, primarily due to restricted instrument mobility and two-dimensional visualization [49,50,51].

### 3.2. Robotic Approach

Robotic surgery, using platforms such as the Da Vinci Xi system, provides high-definition three-dimensional visualization, articulated instruments, and improved surgeon ergonomics [52,53,54]. These technical advantages facilitate more precise dissection in confined anatomical spaces and may reduce conversion rates to open surgery [55,56,57].

Recent comparative studies between robotic and laparoscopic colectomy have reported similar oncological radicality, with some evidence suggesting higher lymph node yields, as well as reduced transfusion requirements and lower rates of temporary or permanent stoma formation in the robotic group [58,59,60,61].

### 3.3. Clinical and Oncological Outcomes

Comparative analyses indicate that operative time may be longer for robotic procedures, particularly during the initial phase of the learning curve; however, this difference tends to decrease with increasing surgical experience [62,63]. Perioperative morbidity and rates of major complications appear comparable between laparoscopic and robotic approaches [64,65,66,67].

Furthermore, robotic colectomy has been associated with a higher number of retrieved lymph nodes, which may have implications for long-term oncological outcomes, although robust evidence regarding survival benefits remains limited [68,69,70,71].

### 3.4. Advantages and Limitations of Minimally Invasive Approaches

The robotic approach offers clear advantages in cases of deep pelvic tumors and in patients with morbid obesity, owing to enhanced instrument articulation and three-dimensional visualization, which enable precise dissection and improved preservation of autonomic nerve structures [72,73,74]. Additionally, robotic technology may facilitate complete tumor excision, maintenance of negative circumferential margins, and increased lymph node retrieval, potentially allowing for more accurate staging and earlier initiation of adjuvant chemotherapy [75,76,77].

Conversely, robotic colectomy is associated with several limitations, including higher costs, longer operative times during the early learning phase, and the need for substantial training. Prior experience in laparoscopic surgery appears to significantly shorten the learning curve for robotic procedures, leading to reduced operative times and improved clinical outcomes [78,79,80].

Laparoscopic surgery remains the standard approach in many centers due to lower costs and wider availability. However, in rectal tumors or patients with complex anatomy, the limitations of laparoscopic instrumentation may result in higher conversion rates to open surgery, which are associated with increased morbidity and prolonged hospitalization [81,82].

### 3.5. Oncological Considerations and Long-Term Outcomes

Comparative studies between robotic and laparoscopic colectomy for colon cancer suggest that both techniques achieve similar oncological outcomes, with high rates of negative resection margins and adequate lymph node harvest [83]. Some recent meta-analyses indicate that the robotic approach may be associated with a modest increase in lymph node yield, potentially facilitating more accurate staging and improved planning of adjuvant therapy [58,59].

In addition, conversion rates to open surgery appear lower in robotic colectomy, which may contribute to reduced perioperative complications and shorter hospital stays [60,61].

Long-term outcomes, including overall survival and disease-free survival, appear comparable between the two approaches, although most available studies remain limited by relatively small cohort sizes and short follow-up periods [68,69,70,71]. Nevertheless, the short-term benefits and potential oncological advantages support the role of robotic surgery as a promising alternative, particularly in centers with established expertise in minimally invasive techniques [78,79].

Thirteen studies reported a significantly longer operative time for robotic procedures compared to laparoscopic approaches [28,84,85,86,87,88,89,90,91,92,93,94,95].

Five studies demonstrated a significantly lower conversion rate to open surgery in robotic right hemicolectomy compared to laparoscopic surgery [26,84,85,88,90], while no study reported a lower conversion rate in favor of the laparoscopic approach.

Several studies did not predefine the type of anastomosis, thereby allowing an indirect assessment of the influence of surgical approach on anastomotic strategy. When only these studies were considered [26,88,94,95], intracorporeal anastomosis was more frequently performed during robotic right hemicolectomy, whereas extracorporeal anastomosis predominated in laparoscopic procedures.

Notably, in the randomized clinical trial conducted by Park et al. [94], intracorporeal anastomosis was performed in 85.7% of patients in the robotic group compared to 20% in the laparoscopic group.

The number of retrieved lymph nodes varied substantially across studies, likely reflecting differences in the extent of lymphadenectomy, ranging from 11 [92] to 55 [85]. Four studies reported higher lymph node yields in the robotic group [26,84,89,95], whereas one study found higher yields in the laparoscopic group [91].

Following right hemicolectomy, length of hospital stay ranged between 3 days [28] and 8 days [88]. Five studies reported a significantly shorter hospital stay after robotic right hemicolectomy compared to laparoscopic surgery [28,87,88,91,96], while none demonstrated shorter hospitalization in favor of laparoscopy.

Postoperative morbidity ranged from 9.9% [96] to 35% [93]. Significant differences in morbidity between approaches were reported in four studies [26,84,93,96], with two favoring laparoscopy [26,84] and two favoring the robotic approach [93,96].

Postoperative mortality remained below 2.4% [89,96] across all studies, with eight studies reporting zero mortality following robotic right hemicolectomy [84,85,86,87,88,92,93,94].

Farah et al. conducted a large retrospective cohort study including 53,209 patients between 2015 and 2020, published in 2023 [97], using the ACS-NSQIP database. Intraoperative outcomes showed that median operative time was longer in robotic procedures, lymph node yield was higher in the robotic group, conversion rates to open surgery were lower, and transfusion requirements within the first 72 h were comparable between groups. Postoperative outcomes demonstrated similar rates of anastomotic leakage, significantly lower rates of postoperative ileus in the robotic group, comparable overall complication rates, similar major morbidity, and no significant difference in 30-day mortality.

Textbook outcome (TO) is an emerging metric for assessing surgical quality, integrating structural, process, and outcome parameters into a single composite indicator. It has been validated across multiple surgical specialties and demonstrates adequate discriminative ability [98,99]. This composite endpoint incorporates multiple parameters, including complications, length of hospital stay (LOS), reinterventions, and readmissions, thereby providing a comprehensive reflection of perioperative course and optimal surgical outcomes [100].

A study conducted in the United Kingdom [101] retrospectively compared robotic and laparoscopic colorectal procedures performed between February 2020 and October 2022 in a high-volume tertiary center performing over 250 oncological resections annually. These findings suggest that robotic surgery represents a viable and effective alternative to laparoscopy in colorectal surgery, with a potentially shorter learning curve for surgeons experienced in laparoscopic techniques. Nevertheless, further research is required to better define the learning curve associated with robotic surgery.

### 3.6. Strength and Quality of Evidence

Although numerous studies report potential advantages of robotic surgery, including lower conversion rates, reduced intraoperative blood loss, improved lymph node harvest, and enhanced short-term recovery, much of this evidence originates from retrospective cohort studies, national database analyses, and propensity score-matched investigations. While these methodologies can reduce selection bias, they cannot fully eliminate residual confounding factors related to surgeon experience, institutional volume, patient selection, and perioperative management protocols. In contrast, randomized controlled trials remain relatively scarce and generally demonstrate comparable oncological and perioperative outcomes between robotic and laparoscopic approaches, with robotic surgery mainly showing technical advantages in specific subgroups such as obese patients, and complex colorectal resections. Therefore, although current evidence supports the safety and feasibility of robotic colorectal surgery, the magnitude of its clinical superiority remains uncertain. Future multicenter randomized trials and standardized reporting of outcomes are needed to better define the true benefits, limitations, and cost-effectiveness of robotic approaches in colorectal cancer surgery.

To improve the synthesis of the available evidence, the principal comparative studies evaluating robotic and laparoscopic colectomy are summarized in Table 2, while the main perioperative and oncological outcomes reported in the literature are presented in Table 3.

## 4. Discussion

The integration of robotic surgery into clinical practice has introduced a high level of precision and technical consistency in the management of colon tumors. Although associated with higher initial costs and, particularly during the early phase of adoption, longer operative times, the potential patient-centered benefits—including reduced transfusion requirements, shorter hospital stay, and decreased need for stoma formation—suggest a meaningful added value [72,73,74,75,76,77].

Moreover, accumulating evidence indicates that robotic surgery may reduce inter-operator technical variability, largely due to standardized instrumentation and enhanced visual feedback [73,74]. This aspect may be particularly relevant in high-volume centers, where procedural standardization can contribute to improved oncological and functional outcomes [75,76,77].

Based on the results of the COLOR, UK MRC CLASICC, and EnROL trials [47,103,104,105,106], professional societies recommend, whenever feasible, the use of minimally invasive approaches over open surgery for right hemicolectomy [104,105]. Currently, laparoscopy represents the standard approach for minimally invasive right hemicolectomy, accounting for 90.5% of cases in Denmark [26], 96.2% in the United States [107], and 97.1% in Australia [96]. However, laparoscopy remains subject to several inherent technical limitations that may impact outcomes in patients undergoing right hemicolectomy.

For instance, central lymphadenectomy at the level of the middle colic artery is technically demanding when performed laparoscopically and may require extension of the extraction incision [84], leading to the concept of laparoscopic-assisted right hemicolectomy [108]. Furthermore, laparoscopic instrumentation—lacking articulation—may limit precise dissection around vascular pedicles at their origin, potentially resulting in suboptimal lymphadenectomy, particularly at the ileocolic pedicle.

Suboptimal lymph node dissection may adversely affect survival, as inadequate staging could lead to under-treatment, with some patients not receiving appropriate adjuvant chemotherapy. In this context, analysis of the Danish Colorectal Cancer Group database (2008–2011) demonstrated suboptimal 4-year disease-free survival in TNM stage I–II right-sided colon cancer following laparoscopic surgery [109], which contributed to the development of the concept of complete mesocolic excision (CME) as a corrective strategy.

Additionally, analysis of the same national database (2014–2018) revealed that 93.6% of patients undergoing laparoscopic right hemicolectomy received an extracorporeal anastomosis [26], with a proportion of 68.7% reported in the MERCY study [110].

Extracorporeal anastomosis, however, typically requires a midline incision and extensive mobilization of the mesentery to approximate bowel ends, thereby exposing patients to increased risks of postoperative pain, wound infection, and postoperative ileus, potentially prolonging hospital stay compared to intracorporeal techniques [108,111,112,113,114,115,116,117,118,119].

The relatively low adoption of intracorporeal anastomosis in minimally invasive right hemicolectomy is largely attributable to its technical complexity when performed laparoscopically. As a consequence, a retrospective cohort study from the Cleveland Clinic reported that a midline extraction site was used in 88.7% of laparoscopic right hemicolectomies, while Pfannenstiel incisions were employed in only a minority of cases [120]. This preference for midline extraction—often associated with extracorporeal anastomosis—has been confirmed by several other studies [112,113,121,122,123], exposing patients to a higher risk of wound-related complications, particularly incisional hernia.

Indeed, a systematic review and meta-analysis reported a cumulative incidence of incisional hernia of 10.6% for midline incisions compared to 0.9% for Pfannenstiel incisions following laparoscopic colorectal surgery [124].

Following the publication of multiple trials demonstrating comparable oncological outcomes and improved postoperative recovery with minimally invasive right hemicolectomy [47,101,104,105,106], laparoscopy has become the standard approach [26,96,107]. Currently, laparoscopic right hemicolectomy typically involves laparoscopic mobilization of the colon, extracorporeal vessel ligation and lymphadenectomy (at least at the ileocolic level), extracorporeal anastomosis, and specimen extraction through a midline incision [26,84,110,112,113,120,121,122,123].

From a theoretical standpoint, a fully minimally invasive right hemicolectomy should include intracorporeal vascular division, lymphadenectomy, and anastomosis, thereby allowing specimen extraction through a Pfannenstiel incision. While technically feasible laparoscopically, this approach has not been widely adopted due to inherent technical constraints. In this context, robotic platforms—offering enhanced dexterity and control—may enable optimization of surgical technique and improved postoperative outcomes.

In our systematic review, 16 studies comparing laparoscopic and robotic right hemicolectomy were identified [26,28,84,85,86,87,88,89,90,91,92,93,94,95,96,125]. These studies consistently reported reduced intraoperative blood loss, lower postoperative complication rates, faster recovery of bowel function, fewer conversions to open surgery, and shorter hospital stay in patients undergoing robotic surgery, at the expense of slightly longer operative times [102,126,127,128].

The improved postoperative outcomes observed with robotic surgery may be largely attributed to the facilitation of intracorporeal anastomosis, which remains technically demanding in laparoscopy. Several studies have reported a higher proportion of intracorporeal anastomoses in robotic compared to laparoscopic approaches [94,95,110,120,129].

In the only randomized controlled trial available, intracorporeal anastomosis was performed in 85.7% of robotic cases compared to 20% in the laparoscopic group [94], likely influencing the choice of extraction site, with a preference for Pfannenstiel incisions in these patients [123]. Similarly, in the MIRCAST study, Pfannenstiel extraction was more frequently associated with intracorporeal anastomosis [95].

Nevertheless, most available studies are subject to potential bias, as they are predominantly observational, include heterogeneous patient populations, and involve variability in surgical techniques (with or without intracorporeal anastomosis, D3 lymphadenectomy, and/or CME). For example, in the MIRCAST study, D3 lymphadenectomy was more frequently performed in the robotic group [95], potentially confounding comparisons by prolonging operative time.

Furthermore, many studies are limited by small sample sizes, database-driven analyses, and lack of randomization. High-quality randomized controlled trials are therefore required to validate these preliminary findings.

To date, only one randomized controlled trial has directly compared robotic and laparoscopic right hemicolectomy [94]. No significant differences were observed in time to first flatus, length of hospital stay, complication rates, postoperative pain, or lymph node yield. Long-term analysis demonstrated comparable survival outcomes between the two approaches [130].

However, a key limitation of this trial was the lack of standardization of the anastomotic technique, as patients in the robotic group could undergo either intracorporeal or extracorporeal anastomosis. Consequently, the potential advantage of robotic surgery—namely the facilitation of intracorporeal anastomosis [128]—was not adequately assessed.

Importantly, intracorporeal anastomosis was performed more frequently in robotic procedures, suggesting that robotic platforms facilitate this technique—a finding supported by systematic review and meta-analysis [128].

The true benefits of robotic right hemicolectomy may therefore be realized primarily in the context of a fully robotic minimally invasive approach incorporating intracorporeal anastomosis. A propensity score-matched analysis (192 patients) comparing fully robotic right hemicolectomy (intracorporeal anastomosis) with robotic-assisted surgery (extracorporeal anastomosis) demonstrated superior postoperative outcomes in terms of pain and bowel function recovery for the fully robotic approach [131].

In a prospective cohort study evaluating fully robotic right hemicolectomy [132], intracorporeal anastomosis was successfully achieved in all patients, with a low conversion rate (3.3%) and a mean operative time of 200.4 ± 114.9 min, which compares favorably with previously reported durations of up to 279 min and 327.5 min in the literature [133]. The study also reported a mean lymph node yield of 22.4 ± 7.6, a mean hospital stay of 5.4 ± 3.8 days, a postoperative morbidity rate of 11.7%, and no mortality.

Despite these promising results, national audit data indicate that intracorporeal anastomosis is not yet routinely performed in robotic right hemicolectomy [26]. Moreover, this approach must be compared against the current standard—laparoscopic right hemicolectomy with extracorporeal anastomosis—and long-term outcomes, including incisional hernia rates influenced by extraction site, remain to be fully evaluated.

Surgeon experience on the robotic platform is also a critical factor, as operative time and conversion rates have been shown to correlate inversely with individual case volume [134].

In the United States, colorectal resections are among the most commonly performed surgical procedures, and robotic surgery is increasingly adopted for CRC. However, evidence supporting a definitive transition from conventional laparoscopy remains limited. While previous studies have reported favorable outcomes for robotic right and left colectomy—such as reduced blood loss, lower conversion rates, and shorter hospital stay—robotic low anterior resection (LAR) has yielded mixed results. Some studies report reduced conversion rates and shorter LOS, whereas others do not demonstrate clear advantages in severe complication rates. Notably, robotic LAR may be associated with higher major postoperative morbidity without significant differences in textbook outcomes [97].

Recent studies have increasingly utilized the NSQIP database to evaluate perioperative outcomes of minimally invasive colorectal surgery [135]. El Aziz et al. highlighted the growing adoption of robotic colorectal surgery and its implications on perioperative outcomes, although their analysis combined right, left colectomies and LAR [136]. Similarly, Soliman et al. compared laparoscopic and robotic approaches for CRC and chronic diverticulitis using NSQIP data [135].

A systematic review and meta-analysis by Tschann et al. demonstrated improved perioperative outcomes for robotic right colectomy, including reduced blood loss, lower conversion rates, and shorter hospital stay [102]. Another meta-analysis by Solaini et al. concluded that robotic left colectomy is non-inferior to laparoscopy in terms of postoperative complications and mortality [9]. Consistent findings suggest improved textbook outcomes, shorter hospital stay, lower conversion rates, and reduced postoperative ileus with robotic right colectomy [137].

In contrast, for left colectomy, although some analyses report lower complication rates with robotic surgery [9], these findings are not consistently reproduced in malignant subgroups. Operative time remains longer in robotic procedures, while conversion rates are lower—findings consistent with our analysis, which demonstrated comparable perioperative morbidity and mortality between approaches, but improved textbook outcomes with robotic surgery.

We hypothesize that robotic surgery may improve outcomes in both right and left colectomy through enhanced three-dimensional visualization, increased instrument dexterity, and the ability to perform complex maneuvers with precision in confined anatomical spaces.

Robotic LAR remains technically more demanding, with an estimated learning curve of 55–65 cases compared to 35–45 for left colectomy and 16–25 for right colectomy. A propensity-matched analysis by Matsuyama et al. demonstrated improved perioperative outcomes with robotic LAR, including lower conversion rates, shorter hospital stay, comparable complication rates, and lower mortality [73]. Similarly, a meta-analysis by Sun et al. reported shorter hospital stay, lower conversion rates, and reduced overall complications with robotic LAR [138].

In the ROLARR trial, Jayne et al. reported conversion rates of 8.1% for robotic and 12.2% for laparoscopic LAR, although the difference was not statistically significant [36]. However, sensitivity analysis suggested lower conversion rates with robotic surgery when performed by experienced surgeons. Our data similarly indicate a higher conversion rate for laparoscopic LAR (10.4%) compared to robotic LAR (3.9%).

A recent multicenter study by Feng et al. demonstrated improved postoperative recovery with robotic surgery for mid and low rectal cancer [41], particularly in anatomically confined pelvic spaces. In our study, the LAR group included tumors of varying levels, as NSQIP data do not provide tumor distance from the anal verge.

Robotic surgery has gained substantial popularity, particularly in rectal and pelvic surgery, due to improved visualization, articulation, precision, and ergonomic advantages [139]. A modest but consistent increase in lymph node yield has also been reported, although survival benefits remain to be confirmed [26,40,140,141,142,143,144].

Despite being in an early adoption phase, current evidence suggests that robotic colorectal surgery is both safe and feasible, with outcomes comparable to established laparoscopic techniques [145,146,147]. Importantly, although operative times may be longer for non-pelvic robotic procedures, this does not appear to translate into increased thromboembolic events, conversion rates, perioperative morbidity, reinterventions, or mortality [148,149].

The limitations inherent to retrospective analyses must be acknowledged, including potential selection bias and the influence of the learning curve, particularly given the inclusion of early robotic cases. Variability in operative setup and docking times further reflects the evolving nature of this technology [150,151].

Notably, results from large series conducted in general hospitals appear consistent with those from high-volume tertiary centers, supporting the feasibility and reproducibility of robotic techniques in appropriately trained settings [101].

With increasing experience, operative times are expected to decrease and align more closely with laparoscopic benchmarks. Evidence suggests a shorter and more favorable learning curve for robotic surgery among surgeons with prior minimally invasive experience, with operative efficiency improving as case volume increases [101,152]. Consequently, high procedural volume and standardized workflows may contribute to reducing the overall cost burden associated with robotic surgery over time [153].

### 4.1. Limitations

The interpretation of the current evidence is further complicated by the inclusion of heterogeneous colorectal procedures within many studies and meta-analyses. Right-sided colectomies, left-sided resections, sigmoid colectomies, and low anterior resections represent distinct surgical entities with different anatomical challenges and perioperative risk profiles. Since the potential advantages of robotic surgery are likely procedure-dependent, particularly in technically demanding pelvic operations, combining these procedures may obscure clinically relevant differences. Second, much of the available evidence is derived from retrospective analyses, which are inherently subject to selection bias and residual confounding. Learning-curve effects may also have influenced reported outcomes, particularly in studies evaluating early experiences with robotic surgery. In addition, publication bias cannot be excluded, as studies reporting favorable outcomes are more likely to be published. Finally, long-term oncological data remain limited, and further prospective studies with extended follow-up are needed to better define the comparative effectiveness of robotic and laparoscopic approaches. Therefore, caution is warranted when extrapolating pooled results, and future research should focus on procedure-specific analyses with standardized outcome reporting.

### 4.2. Economic Considerations and Cost-Effectiveness

One of the principal limitations of robotic colorectal surgery remains its higher cost compared with conventional laparoscopy. Several studies have demonstrated significantly higher direct hospital costs for robotic procedures, even when perioperative outcomes are comparable to laparoscopic surgery. However, cost-effectiveness analyses have produced heterogeneous results. Some authors suggest that the higher initial costs may be partially offset by lower conversion rates, reduced postoperative complications, shorter hospital stays, and faster recovery, particularly in technically demanding procedures and high-risk patient populations. Nevertheless, the current evidence remains insufficient to conclusively demonstrate the economic superiority of robotic colectomy, and cost-effectiveness appears highly dependent on local healthcare systems, reimbursement policies, and procedural volume. Further prospective studies incorporating long-term clinical outcomes and quality-adjusted life-year analyses are needed to better define the economic value of robotic colorectal surgery.

### 4.3. Future Research Directions

Further research is needed to clarify the long-term benefits of robotic surgery in colorectal cancer, particularly regarding overall survival, disease-free survival, and recurrence rates. Future studies should also place greater emphasis on patient-reported outcomes, including quality of life and functional recovery. In addition, robust cost-effectiveness analyses are required to determine whether the potential clinical advantages of robotic surgery justify its increased financial burden. Addressing these issues through large multicenter studies will help define the optimal role of robotic surgery in colorectal cancer treatment.

## 5. Conclusions

Based on the current evidence, robotic procedures can represent a safe and effective alternative to laparoscopic ones in the management of colorectal cancer. It offers potential advantages in lymph node harvest, earlier initiation of adjuvant chemotherapy, and improved short-term outcomes. However, higher costs and the need for a structured learning curve must be carefully considered, and the choice of surgical approach should be individualized based on patient characteristics and surgical expertise.

The present review of predominantly non-randomized studies suggests that robotic surgery may provide superior postoperative outcomes following right hemicolectomy, particularly through facilitating intracorporeal anastomosis, enabling off-midline specimen extraction, and supporting extended lymphadenectomy.

Ongoing randomized trials, such as PRORHEM and ROLACART-1 pilot studies, are expected to further clarify these findings. Robotic right and left colectomy appear to improve textbook outcomes and reduce conversion rates, while maintaining comparable morbidity and mortality to laparoscopy.

In contrast, robotic LAR demonstrates similar textbook outcomes but may be associated with higher rates of anastomotic leakage, postoperative ileus, and major morbidity—likely reflecting procedural complexity, longer learning curves, and potential selection bias.

Given the increased financial cost of robotic surgery, its broader adoption should be guided by robust evidence. The associations identified in this study should be carefully considered in surgical decision-making for patients with colorectal cancer.

Future research should focus on long-term oncological outcomes, cost-effectiveness, quality of life, and the optimization of robotic integration into routine clinical practice.

## Figures and Tables

**Table 1:** Literature Search and Study Selection Process According to PRISMA 2020.

PRISMA Phase	Selection Process	n
Identification	Records identified through database searching	250
	Duplicate records removed	Not reported
Screening	Records screened (title and abstract review)	250
	Records excluded	50
Eligibility	Full-text articles assessed for eligibility	200
	Full-text articles excluded	30
Included	Studies eligible for inclusion	170
	Studies excluded after quality assessment and relevance evaluation	20
	Studies included in the final review	150

**Table 2:** Selected Studies Comparing Robotic and Laparoscopic Colectomy.

Study	Design	Sample Size	Main Findings Favoring Robotic Surgery	Main Limitations
Park et al. [94]	Randomized controlled trial	Not specified	Higher rate of intracorporeal anastomosis (85.7% vs. 20%)	Single-center experience
Farah et al. [97]	Retrospective ACS-NSQIP analysis	53,209 patients	Lower conversion rate, higher lymph node yield, lower postoperative ileus	Retrospective database study
Spinoglio et al. [90]	Comparative cohort	Not specified	Lower conversion rate, higher lymph node harvest	Non-randomized design
Alawadi et al. [85]	Comparative cohort	Not specified	Lower conversion rate, greater lymph node yield	Selection bias possible
Siani et al. [88]	Comparative study	Not specified	Lower conversion rate and shorter hospital stay	Limited sample size
Dolejs et al. [28]	Retrospective cohort	Not specified	Higher lymph node retrieval	Retrospective design
Solaini et al. [89]	Comparative cohort	Not specified	Lower conversion to open surgery	Single-center study
Kim et al. [71]	Comparative cohort	Not specified	Shorter hospital stay with robotic approach	Higher lymph node yield favored laparoscopy
Tschann et al. [102]	Comparative study	Not specified	Lower postoperative morbidity	Limited follow-up

**Table 3:** Summary of Outcomes Reported in the Literature.

Outcome	Robotic Surgery	Laparoscopic Surgery	Strength of Evidence
Conversion to open surgery	Lower in most studies	Higher	Moderate
Operative time	Longer	Shorter	High
Blood loss	Slightly lower	Slightly higher	Moderate
Lymph node yield	Often higher	Comparable	Moderate
Length of hospital stay	Shorter in several studies	Comparable/slightly longer	Moderate
Postoperative morbidity	Similar or slightly lower	Similar	Moderate
30-day mortality	Comparable	Comparable	High
Overall survival	Comparable	Comparable	Low–Moderate
Disease-free survival	Comparable	Comparable	Low–Moderate
Cost	Higher	Lower	High

## Data Availability

No new data were created or analyzed in this study. Data sharing is not applicable to this article.
